# Exploratory Amplicon-Based Metagenomic Profiling and Chemical Fingerprinting of Three Spontaneously Fermented Southern African Traditional Products

**DOI:** 10.3390/foods15213935

**Published:** 2026-11-03

**Authors:** Mbuyane Lethiwe Lynett, Chigwada Aubrey Dickson, Tekere Memory

**Affiliations:** 1 Department of Life and Consumer Sciences, College of Agriculture and Environmental Sciences, University of South Africa (UNISA), Science Campus, Roodepoort 1709, South Africa; 2 Department of Environmental Sciences, College of Agriculture and Environmental Sciences, University of South Africa (UNISA), Science Campus, Roodepoort 1709, South Africa

**Keywords:** spontaneous fermentation, 16SrRNA and ITS amplicon sequencing, lactic acid bacteria, fungi, substrate-driven diversity

## Abstract

Traditional fermented foods are integral to African culinary heritage and nutrition, yet their microbial ecology remains underexplored in many regions. This study employed amplicon sequencing (16S rRNA and ITS) and Fourier Transform Infrared (FTIR) spectroscopy to characterize the microbial communities and biochemical profiles of spontaneously fermented cow’s milk (amasi), sorghum–maize slurry (ting-type gruel), and sour maize gruel (mahewu). Amasi displayed the highest diversity, dominated by *Lactococcus* and *Malassezia restricta*. Mahewu was dominated by *Clostridium sensu stricto* and *Malassezia restricta*. The ting-variant showed low diversity with *Weissella* dominance and *Symbiotaphrina* species. FTIR spectra corroborated these patterns, highlighting lipid/protein bands in amasi and carbohydrate/organic acid signatures in the fermented cereal products. Beta-diversity analyses confirmed strong substrate-driven separation, with limited shared taxa underscoring ecological specialization. These findings reveal how raw material composition and processing shape spontaneous fermentation outcomes, while highlighting potential safety risks from opportunistic taxa and opportunities for probiotic strain isolation from beneficial taxa. Future studies are required to determine the viability and characteristics of the microbial flora in these fermented beverages to determine if their occurrence is significant. The results in this study show that metagenomic and FT-IR spectroscopy can be used to profile traditional fermented products, but this must be coupled with in-depth metabolite analyses using quantitative instruments and machine learning technologies for predictive fingerprinting to determine the identity, safety, and functionality of these culturally significant foods.

## 1. Introduction

Fermentation is the transformation of a substrate into a new product via the action of microorganisms, and this process has been used for centuries in Africa for food preservation [1,2,3,4,5,6]. Local crops such as maize and sorghum were fermented into a myriad of products (porridge, beer, non-/low-alcoholic beverages, etc.) for nutrition as well as enjoyment [7,8,9]. Fermented food products are a significant part of African culinary heritage because they have long been used in traditional ceremonies such as weddings, funerals, and initiation rites [10,11]. In contemporary settings, fermentation technology is still applied to produce food products with increased nutritional value, favorable organoleptic properties, and for preservation purposes, especially in low-income areas [12,13,14,15,16].

Traditional fermented foods contain microorganisms that produce metabolites that inhibit pathogenic microorganisms, and improve product safety and shelf-life [17,18]. Indeed, the production of organic acids by lactic acid bacteria or the production of acetic acid from *Acetobacter* sp. decreases pH and inhibits pathogenic microorganisms that are sensitive to acidic environments [19,20,21]. The conversion of sugars in substrates such as palm sap or sorghum by yeasts into ethanol is also inhibitory to ethanol-sensitive contaminating microorganisms that are a health risk [22,23,24]. Additionally, selected fermentative microorganisms in African traditional foods are also known to secrete antimicrobial proteins, promote food digestibility through the breakdown of complex carbohydrates, and exhibit probiotic characteristics [18,25,26,27,28].

Although traditional fermented foods may contain beneficial flora, the manufacturing of these products is difficult to manage due to the utilization of spontaneous fermentation technology with microorganisms from the substrate, surrounding environment, and vessel driving the process [15,29,30,31]. Alternatively, back-slopping (the use of a previously fermented batch as starter culture) is used in some communities [32,33]. Poor safety practices also result in contamination with pathogenic bacteria such as *Stapyhlococcus aureus*, *Escherichia coli*, Enterobacteriaceae, and opportunistic pathogenic yeasts from the *Candida* genus, which are prevalent in fermented food products in Africa [18,34,35,36]. Furthermore, contamination by mycotoxins due to the presence of fungi such as *Aspergillus* sp. and *Fusarium* sp. is also detected in traditional fermented products [37,38,39]. The presence of pathogens and hazardous toxins in fermented foods and beverages is particularly concerning, as these products are also sold by small-scale vendors in the informal sector [16].

In Southern Africa, the manufacturing of some products such as amasi (traditional fermented cow milk) and mahewu (fermented maize gruel) is widely commercialized [40]. However, traditional fermented products are still consumed in rural areas as well as during cultural ceremonies using traditional processing methods, which include spontaneous fermentations. Therefore, there is a need to further survey the microbiome of traditional Southern African fermented products to understand what consumers are ingesting and to profile microbial communities driving the spontaneous fermentation process. This study aims to assess the microbial profile of three Southern African traditional fermented products, namely amasi (fermented cow milk), a ting-type of cereal gruel (sorghum- and maize-meal-based product) and mahewu (maize-based cereal gruel) using metagenomics.

## 2. Materials and Methods

### 2.1. Preparation of Traditional Fermented Products

Three traditional Southern African fermented products, amasi (spontaneously fermented cow’s milk), mahewu (fermented maize gruel), and a ting-type cereal gruel (sorghum- and maize-meal-based slurry), were prepared under controlled laboratory conditions following traditional practices with minor adaptations. For amasi, fresh raw cow’s milk was obtained from a local dairy farm in Roodepoort, Gauteng, South Africa. A 350 mL aliquot was transferred into a sterile 500 mL Erlenmeyer flask and incubated statically at 30 ± 1 °C for 48 h to allow spontaneous fermentation, mimicking traditional household preparation [41,42]. For mahewu, 28 g of commercial maize meal was suspended in 350 mL potable water in a sterile 500 mL Erlenmeyer flask. The mixture was boiled for 15 min with continuous stirring to gelatinize the starch, cooled to ~40 °C, and supplemented with 2 g wheat flour as a traditional starter source [43,44]. The slurry was then incubated statically at 30 ± 1 °C for 48 h. For the ting-variant cereal gruel, adjustments were made in processing. Approximately 25 g of sorghum meal and 50 g maize meal were mixed with 350 mL potable water in a sterile 500 mL Erlenmeyer flask and fermented spontaneously at 30 ± 1 °C for 24 h [45]. The mixture was then boiled for 15 min, adjusted to a thick porridge consistency by adding water, supplemented with ~25 g fresh sorghum meal, and further fermented at 30 ± 1 °C for 48 h. At the end of the 48 h incubation period, samples were aseptically collected, aliquoted, and immediately stored at −20 °C until further analysis.

### 2.2. Determination of pH

The pH of each fermented product was measured using a calibrated digital pH meter (model HI 2211, Hanna Instruments, Woonsocket, RI, USA) at 25 °C according to standard methods.

### 2.3. Fourier Transform Infrared (FTIR) Spectroscopy

FTIR analysis was performed to characterize the biochemical composition of the fermented products. Approximately 20 µL of each homogenized sample was applied directly onto the diamond attenuated total reflectance (ATR) crystal of a Nicolet iS5 FTIR spectrometer (Thermo Fisher Scientific, Waltham, MA, USA) equipped with a deuterated triglycine sulfate (DTGS) detector. Spectra were acquired in the mid-infrared range (4000–500 cm^−1^) at a resolution of 4 cm^−1^, with 16 co-added scans per sample to improve signal-to-noise ratio. Background spectra of the clean ATR crystal were collected before each measurement and automatically subtracted. Raw spectra were subsequently preprocessed in OriginPro 2023 using standard procedures: baseline correction (rubberband algorithm), Savitzky–Golay smoothing (second-order polynomial, 17-point window) for noise removal, and standard normal variate (SNV) normalization. Outlier detection was performed by principal component analysis (PCA) of the spectral matrix using Hotelling’s T^2^ and Q residual statistics at the 95% confidence level; no spectra were identified as statistical outliers. For future extensions involving hyperspectral- or imaging-based FTIR data, more advanced denoising strategies such as PCA combined with wavelet shrinkage [46] or denoising convolutional autoencoders [47] may offer superior SNR enhancement. Spectra were exported as ASCII files for subsequent analysis.

### 2.4. Genomic DNA Extraction

Total genomic DNA was extracted from 1 mL aliquots of each fermented sample using the NucleoSpin^®^ Microbial DNA Kit (Macherey-Nagel, Düren, Germany) according to the manufacturer’s protocol. To ensure efficient lysis of Gram-positive lactic acid bacteria and yeasts with recalcitrant cell walls that are common in these fermented substrates, the manufacturer-recommended bead-beating duration was extended from 5 min to 10 min at maximum speed (6.5 m s^−1^) using a FastPrep-24 bead beater equipped with Lysing Matrix E tubes. Extractions were performed in triplicate per sample to account for potential extraction bias. Triplicate extracts were pooled to obtain a representative DNA pool per product. DNA concentration and purity were quantified using a Qubit^®^ 4 Fluorometer with the Qubit dsDNA HS Assay Kit (Thermo Fisher Scientific, Waltham, MA, USA). Only high-quality DNA preparations (concentration 20–150 ng µL^−1^; A260/A280 ratio 1.8–2.0) were used for downstream sequencing [48].

#### 2.4.1. Library Preparation and PacBio SMRT Sequencing

Long-read amplicon sequencing of the fungal internal transcribed spacer (ITS) region and bacterial 16S rRNA gene was performed using Pacific Biosciences (PacBio) single-molecule real-time (SMRT) sequencing technology [48]. Barcoded universal primers were employed to generate amplicon libraries following the PacBio recommended protocol for barcoded SMRTbell libraries [48]. For fungal profiling, the ITS1F/LR5 primer pair was used to amplify the ITS1–5.8S–ITS2–LSU partial region [49]. Bacterial 16S rRNA gene libraries targeted the V1–V9 region using barcoded 27F/1492R primers [50]. Sequencing was conducted on a PacBio RSII instrument using P6-C4 chemistry with 360 min movie times at Inqaba Biotec (Pretoria, South Africa). Raw sequencing deposited in the NCBI Sequence Read Archive (SRA) will be made available upon request.

#### 2.4.2. Bioinformatics and Data Analysis

Raw subreads were processed using the Quantitative Insights into Microbial Ecology 2 (QIIME2, version 2023.9) pipeline on the Nephele cloud platform [48]. Demultiplexing, quality filtering, denoising, and chimera removal were performed with DADA2 [51]. Amplicon sequence variants (ASVs) were taxonomically classified against the UNITE database (version 9.0) for ITS sequences [52] and the SILVA database (version 138.1) for 16S rRNA sequences [50] using the naive Bayes classifier implemented in QIIME2. Alpha-diversity (Shannon index, observed ASVs) was calculated after rarefying each library to the minimum number of high-quality reads retained across the three samples, and beta diversity (Bray–Curtis dissimilarity) metrics were calculated and visualized using principal coordinate analysis (PCoA). Statistical comparisons were performed in R (version 4.3.1) using the vegan and phyloseq packages [53,54]. Figures were generated using OriginPro 2023 (OriginLab Corporation, Northampton, MA, USA) and ggplot2 [55]. All steps were performed in compliance with MIxS standards for amplicon sequencing data [56]. PICRUSt2 was used to generate functional predictions inferred from the 16S rRNA gene [57].

## 3. Results and Discussion

### 3.1. pH Analysis

Organic acids such as lactate, acetate, and butanoate produced during fermentation impact the sensory properties of food as well as the microbial load [58,59,60]. Thus, the acidity of fermented products is routinely monitored as a quality indicator. In this study, the acidity of the fermented products was assessed via pH measurements (Table 1). The pH of the amasi in this study was 4.348 and aligns with previous studies, which reported pH values that range between 4 and 5.4 [41]. The pH of the mahewu in this study was 4.005, aligning with a previous report from Swaziland whereby the pH of mahewu samples ranged between 2.95 and 4.51 [20]. The pH of optimized mahewu produced in South Africa from maize ranged from 3.50 to 4.20 [43]. In this study, a pH value of 3.451 was observed for the cooked ting-variant, also aligning with previous studies, whereby a pH of 3.5 was reported for motoho (sorghum slurry) in South Africa [61]. The pH of a maize–sorghum fermented gruel from Tanzania was similar to the ting-variant [62]. However, another study in South Africa reported high pH levels (5–6) and attributed this uncharacteristically high pH to the presence of bioactive compounds in the seed coat of the grain with may impede fermentation [63]. To generate a valid chemical fingerprint of these fermented products and develop predictive models, pH analyses coupled with titratable acidity measurements have to be repetitively analyzed under different conditions to develop a robust dataset [45,64,65,66].

### 3.2. Fourier Transform–Infrared Spectroscopy (FTIR)

Figure 1 shows that the FTIR spectrum of amasi (A) exhibited a broad absorption band at ~3500–3300 cm^−1^, attributed to O–H stretching vibrations primarily from water and N–H stretching from proteins [17,67]. This region is characteristic of fermented dairy products, where residual moisture and amide groups in milk proteins (caseins, whey) dominate. Sharp peaks at ~2920 cm^−1^ and ~2850 cm^−1^ correspond to asymmetric and symmetric C–H stretching of alkyl chains, indicative of lipids and fatty acids present in milk fat [67]. These bands are consistent with the high lipid content typical of cow’s milk and are frequently observed in FTIR spectra of traditional fermented milk [41,68]. A prominent absorption between 1700 and 1600 cm^−1^ was assigned to the C=O stretching vibration of carboxylic acids, esters, and amide I groups (protein backbone) [67,69]. The shoulder at ~1740 cm^−1^ likely reflects free fatty acids and short-chain organic acids (e.g., lactate, acetate) cereal fermentations [17,20]. The region ~1650–1550 cm^−1^, corresponding to amide II (N–H bending and C–N stretching), infers the presence of milk proteins. A band at ~1400–1200 cm^−1^, attributed to C–O and C–N vibrations, and a fingerprint region (~1200–900 cm^−1^) associated with pyranose ring vibrations of lactose and other carbohydrates, align with residual milk sugars and fermentation-derived metabolites [67,69].The spectra of mahewu (M) and the ting-variant (T) displayed a broad O–H stretching band at ~3400–3200 cm^−1^, primarily arising from water used in the slurry preparation and hydroxyl groups in carbohydrates and fermentation metabolites. Similar broad O–H bands have been reported in FTIR spectra of Ethiopian borde (a fermented sorghum/maize beverage) and Tanzanian togwa (fermented cereal), where they reflect residual moisture and other hydroxyl-containing functional groups [70]. Both cereal-based products exhibited C=O stretching vibrations between 1700 cm^−1^ and 1600 cm^−1^, indicative of esters, aldehydes, and carboxylic acids produced during fermentation [70,71]. These peaks are consistent with the formation of short-chain organic acids (lactic, acetic, propionic acids) by Lactic Acid Bacteria (LAB), which are dominant in maize/sorghum gruels [43,44]. The region ~1200–1000 cm^−1^, showing strong C–O–C and C–OH stretching, corresponds to polysaccharides, residual starch, and oligosaccharides, which are more pronounced in cereal-based ferments due to incomplete starch degradation. The fingerprint region (~1200–900 cm^−1^) further reflects ring vibrations of carbohydrates and fermentation-derived metabolites, aligning with spectra of similar African cereal porridges [70]. Although these spectra reveal substrate-dependent biochemical signatures, the interpretations remain semi-quantitative and future research must include quantitative analyses of metabolites via instruments such as High-Performance Liquid Chromatography for organic acids and sugars, as well as Gas Chromatography with Flame Ionization Detection for volatile aroma compounds.

### 3.3. Community Diversity and Statistical Validation

Sequencing effort and quality control yielded sufficiently high-quality reads for reliable community profiling of amasi, mahewu and the ting-variants (Figure S1). After demultiplexing, quality filtering (Phred ≥ 20 across ≥ 90% of bases), denoising, and chimera removal with DADA2, the final high-quality read counts were: amasi (12,847 bacterial/855 fungal), mahewu (9236 bacterial/221 fungal), and ting-variant (11,305 bacterial/1273 fungal). Alpha-diversity metrics were calculated after rarefying each library to the minimum number of high-quality reads retained across the three samples (9236 reads for bacterial communities and 221 reads for fungal communities) to enable fair comparisons despite modest fungal read recovery in the highly acidic mahewu sample.

Figure S2 shows that the alpha-diversity (Shannon index) varied substantially across products, with amasi exhibiting the highest bacterial diversity (Shannon index = 1.393 ± 0.12), followed by the ting-variant (1.05 ± 0.08) and mahewu showing markedly lower diversity of 0.087 ± 0.03 (values represent means from rarefied data; no formal testing applied due to composited samples). Fungal alpha-diversity followed a similar trend, with amasi richest and mahewu most depauperate. Beta-diversity analysis using Bray–Curtis dissimilarity and PCoA ordination revealed complete separation of bacterial and fungal communities among the three products. Given the experimental design (one composite library per product, derived from biological triplicates), formal multivariate testing, such as PERMANOVA, was underpowered and not performed, as recommended for low-replication exploratory studies to avoid spurious significance [72,73]. However, pairwise Bray–Curtis dissimilarities exceeded 0.95 for all comparisons, and ordination explained >95% of variation on the first two axes, providing strong descriptive evidence of substrate-driven community structuring. This pattern is consistent with comparable metagenomic surveys of African fermented foods, where substrate type consistently accounts for the majority of beta-diversity variance despite limited replication [42,74,75].

### 3.4. A Comparative Analysis of the Bacterial Microbiome of Spontaneously Fermented Traditional Products

The bacterial microbiome of the three spontaneously fermented Southern African products, amasi (milk-based), mahewu (maize-based cereal gruel), and the ting-variant (sorghum- and maize-meal-based cereal gruel), was profiled using metagenomic sequencing of the 16S rRNA gene. The relative abundance of bacterial taxa at the genus level (or lowest confidently assigned taxonomic level) is presented in Figure 2.

*Weissella* dominated the ting-variant (98.4%) fermentation, with minor contributions from *Leuconostoc* (1.5%) and *Cyanobacteria* (0.1%). *Weisella* species was dominant in millet-based fermented porridge from Ghana known as Hausa koko [76]. This genus was also prevalent in motoho (sorghum-based fermented porridge from South Africa), ogi (Nigerian fermented cereal gruel for porridge) made from yellow maize or sorghum, wara (Nigerian soft cheese), and Nigerian fermented milk [74]. *Weissella* spp., *Weissella confusa*, *Weissella cibaria,* and *Weissella cibaria* were also isolated from Zimbabwean mahewu [44]. *Weisella* is prevalent in fermented foods, with some species observed to have probiotic and antimicrobial properties desirable in the food industry and others identified as opportunistic pathogens [77,78]. Species-level resolution is therefore required in future studies to determine whether the *Weissella* strains in this study are predominantly beneficial or potentially pathogenic. The mahewu was characterized by a high abundance of *Clostridium sensu stricto* (78.3%), along with *Bacillus* (13.2%) and *Kosakonia* (4.6%). The *Clostridium sensu stricto* genus contains approximately 118 species isolated from various sources (humans, animals, roots, sludge digestors, fermentation pits, etc.) and is of importance due to their pathogenicity or industrial relevance [79,80]. The prevalence of this genus in this study aligns with previous reports where the *Clostridium sensu stricto* was identified as one of the dominant genera in optimal mahewu produced from white and yellow maize [43]. *Bacillus* is prevalent in fermented foods throughout the globe and is also reported in traditional fermented porridges and gruels from Burkina Faso, Nigeria, South Africa, and Kenya, amongst other countries [81,82,83]. *Kosakonia* is a part of the Enterobacteria group commonly associated with plants and is also reported as a facultative human pathogen [84,85]. *Kosakonia cowanii* was detected in umqombothi (sorghum beer) in South Africa via 16S full-length amplicon sequencing, and *Kosakonia pseudosacchari* sp. nov was identified as an endophyte of *Zea mays* [86,87]. Therefore, the prevalence of the *Kosakonia* genus in mahewu in this study may be due to the utilization of maize as substrate. In amasi, *Lactococcus* was the most abundant genus (50.9%), followed by *Enterococcus* (20.8%), *Streptococcus* (5.6%), *Enterobacter* (5.3%), and *Klebsiella* (4.8%). *Lactococcus* is one of the dominant genera responsible for milk fermentation, with the species *Lactococcus lactis* subsp. *Lactis* widely accepted as a probiotic and starter culture to produce functional foods [88,89,90,91,92]. In Zambia, 168 mabisi (traditional fermented milk) samples were collected across 8 provinces, and *Lactococcus* was identified as the dominant genus, aligning with data in this study [42]. However, in another study, *Lactobacillus* was the dominant genus (13.6%) in South African amasi, followed by *Lactococcus* (7.6%) and *Acinetobacter* sp. (5.6%) [75]. *Lactobacillus* is widely reported in milk fermentation, Generally Regarded As Safe (GRAS), and known for species with probiotic traits with applications in the functional food market [93,94,95,96,97,98,99]. In this study, *Lactobacillus* was present at low levels in amasi (<5%) and absent in the other products. This observation is consistent with comparative metagenomic studies showing that *Lactobacillus* abundance is often higher in artisanal field samples than in laboratory-controlled fermentations [74]. In contrast, the prevalence of Enterococci in milk is high, with species such *as Enterococcus faecalis* and *Enterococcus faecium* prevalent in fermented milk products [100]. The genus *Enterococcus* is controversial because while it contains species with probiotic traits, functional traits, and desirable traits for cheesemaking, it is also reported as an opportunistic pathogen [101,102]. In a previous study, *Enterococcus* was reported to be exclusively in South African raw cow and goat milk (before amasi fermentation), whereas in another, the Enterobacteriaceae family was observed in amasi from South Africa, albeit not dominant [75,103]. In the Zambian fermented milk product known as mabisi, *Enterobacter* was identified as one of the dominant genera [42]. Similarly, *Enterobacter* was dominant in Ethiopian milk and milk products [104]. Thus, the abundance of Enterococcus in this study aligns with previous reports.

### 3.5. Taxonomic Heatmap of Bacterial Communities in Spontaneously Fermented Southern African Products

Figure 3 represents the taxonomic heatmap of bacterial communities in spontaneously fermented products. The heatmap shows that mahewu exhibited the broadest taxonomic spread among the cereal-based products, with detectable read counts for *Pantoea*, *Pseudomonas*, *Lactococcus*, *Streptococcus*, and *Leuconostoc* in addition to the dominant *Clostridium sensu stricto*, *Bacillus*, and *Kosakonia*. *Pantoea* species, members of the *Enterobacteriaceae* commonly associated with plant surfaces and endophytes, have been consistently recovered from traditional African cereal porridges and likely originate from the raw materials [76]. The detection of *Pseudomonas* aligns with previous reports in unpasteurized or minimally processed cereal beverages [105]. Amasi displayed the highest taxonomic richness, uniquely harboring significant read counts of *Enterobacter*, *Enterococcus*, *Escherichia-Shigella*, and *Klebsiella*, all belonging to the Enterobacteriaceae family. This family is frequently detected in raw and spontaneously fermented milk across Southern Africa, often originating from the farm environment, milking equipment, or animal udder microbiota [42,75,103]. The ting-variant exhibited the lowest bacterial diversity, with detectable reads limited to *Weissella*, *Leuconostoc*, and *Cyanobacteria*. The near-exclusive dominance of *Weissella* (log_10_-transformed counts ≈ 1.38) and the virtual absence of Enterobacteriaceae and clostridia underscore the selective pressure exerted by the additional processing step of heating the porridge before the second fermentation step.

### 3.6. Differentiation PCoA Analysis of the Bacterial Communities in Fermented Products

Figure 4a shows that the PCoA ordination demonstrates clear differentiation among the three products. The ting-variant (T) is positioned farthest from the others along PCoA1, consistent with its distinct cereal-based matrix and the dominance of *Weissella* spp. and possible treatment effects due to cooking the cereal prior to further fermentation. Amasi (A) and mahewu (M) are separated primarily along PCoA2, reflecting differences between milk-derived and maize-derived substrates in spontaneous fermentations. This strong beta-diversity pattern aligns with previous culture-independent studies of African fermented foods, where substrate composition (dairy vs. cereal) consistently emerges as the primary driver of microbial community structure [42,43,74,75]. The Venn diagram in Figure 4b further reveals extremely limited taxonomic overlap: only *Weissella* is present in all three products. This finding is consistent with the widespread occurrence of *Weissella* species in both cereal-based [44,76] and milk-based African ferments [42], likely reflecting its ecological versatility and tolerance to a range of fermentation conditions. Amasi contained the highest number of unique taxa, including several Enterobacteriaceae, which are commonly associated with raw milk environments [75,103]. These results highlight that spontaneous fermentation in these traditional products leads to highly substrate-specific bacterial consortia, with minimal shared core microbiota.

#### Predicted Functional Potential of Bacterial Communities

To explore the potential metabolic capabilities associated with the observed taxonomic profiles, PICRUSt2 was used to predict KEGG pathway abundances from the 16S rRNA amplicon data (Figure S2). The functional heatmap (Figure S2a) revealed clear substrate-specific metabolic specialization. The *Clostridium sensu stricto* cluster was highly abundant in mahewu and is known to produce butyric acid (butanoic acid) during fermentation [106,107,108,109]. Thus, the enrichment of the butanoic pathway aligns with the high prevalence of Clostridia in mahewu, low pH, and FT-IR spectra. The enrichment of the glycolytic and pyruvate pathway are indicators of fermentative activity by the microorganisms present in mahewu. The enrichment of glycolysis and pyruvate metabolism observed in amasi aligns with milk fermentation as lactic acid bacteria catabolize lactose into glucose and galactose, resulting in the activation of the glycolytic pathway and the production of pyruvate, which is further broken down into lactic acid as well as other by-products [110,111]. Furthermore, the amino acid enrichment in amasi aligns with milk fermentations, as proteolysis is required for the catabolism of milk proteins into peptides or amino acids that can be used by lactic acid bacteria, and the presence of yeasts can improve LAB access to vital amino acids [112]. The biodiversity in the ting-variant was low, and fermentation was primarily driven by *Weisella* sp., which ferments carbohydrates into lactic acid [113,114,115]. Therefore, the enrichment of the glycolytic and pyruvate pathway (towards lactate production) was due to the prevalence of this lactic acid bacteria in the ting-variant. The stacked bar chart (Figure S2b) further highlights that glycolysis/pyruvate metabolism constituted the largest functional category across all samples, while butyrate-related pathways were most prominent in mahewu. These predicted functional profiles provide additional support for the substrate-driven metabolic differentiation observed in both the taxonomic composition and the FTIR biochemical fingerprints.

### 3.7. Comparative Analysis of the Fungal Microbiome in Spontaneously Fermented Traditional Products

Although lactic acid bacteria dominate non-alcoholic or low-alcohol traditional African fermented foods, fungi are also present [116,117,118]. The fungal communities of the three products, profiled via PacBio long-read ITS sequencing, are shown in Figure 5.

The ting-variant displayed the highest fungal richness and was dominated by *Symbiotaphrina kochii* (43.0%) and *Symbiotaphrina microtheca* (11.8%), followed by *Byssochlamys* species (10.8%) and *Paecilomyces maximus* (10.2%). *Symbiotaphrina* species are obligate symbionts of anobiid beetles (such as *Stegobium paniceum* and *Lasioderma serricorne*) which are pests commonly infesting stored grains, dried foods, and spices [119,120,121,122,123,124]. *Byssochlamys* sp. (11.0%) was also identified in the ting-variant, and this fungus is reported as a contaminant of fruit juices, concentrates, as well as grains, while in other studies, it is reported to produce pectinases desirable for applications in the food industry [125,126]. Although the ting-variant was cooked before fermentation, *Byssochlamys* sp was still present because it is a heat-tolerant mold [125]. *P. maximus* (10.5%) was detected in the ting-variant aligning with the previous literature whereby the thermotolerant *Paecilomyces* genus was detected in maize and sorghum products [127,128,129]. Selected species from the genus are also opportunistic pathogens; thus, further research is required to assess the fungal profile of indigenous fermented foods to prevent harm [130]. Amasi is a milk-based product of bacterial fermentation, and the prevalence of fungi is often attributed to contamination from raw milk, the surrounding environment, and fermentation vessels. In this study, 920 total reads were observed in amasi, showing a balanced distribution with *M. restricta* contributing 26.3%, *Coriolopsis hainanensis* contributing 22.9%, *Byssochlamys* sp. contributing 15.2%, and *P. maximus* contributing 15%, reflecting a diverse fungal profile. In a previous study, *M. restricta* was identified as one of the common yeast species in amasi produced from cow and sheep milk, but the prevalence of *C. hainanensis*, a saprophyte recently discovered in China, is underreported in African traditional fermented beverages [75,131]. The presence of *Byssochlamys* and *Paecilomyces* species in amasi aligns with the previous literature, whereby these fungi were isolated in milk-based products [128]. Mahewu, with only 221 reads, was heavily dominated by *M. restricta* at 80.1%, with minor contributions from *Candida albicans* (4.5%) and *Byssochlamys* sp., suggesting a highly selective environment. Reports on the prevalence of *M. restricta* in mahewu are limited, but in a previous study, this fungus was identified for the first time in a Ghanian millet-based fermented porridge known as hausa koko and was reported to drive fungal diversity during fermentation [76]. The genus *Candida* is detected in spontaneously fermented traditional products such as sorghum beer, palm wine, and porridge-like products such as mahewu [132,133,134,135]. In a previous study, this yeast was isolated from a fermented cereal known as fura from West Africa and showed pathogenic traits, as hyphal growth was observed on mucosal cell lines [15]. *Candida albicans* is the most clinically relevant pathogen known to infect immunocompromised individuals andits presence in mahewu in this study is concerning.

### 3.8. Taxonomic Heatmap of Fungal Communities in Spontaneously Fermented Southern African Products

The taxonomic heatmap of log_10_-transformed ITS read counts in Figure 6 provides a complementary view of fungal distribution, emphasizing presence and relative enrichment across the three products. The heatmap shows a high enrichment of *Symbiotaphrina kochi*, followed by *Symbiotriphina microtheca* and *Paecilomyces maximus* and *Byssochlamys* sp. in the ting-variant, with the latter two fungi being equally prevalent. Next, *Xeromyces bisporus* and *Malasseiza restrica* were equally abundant. The presence of *X. bisporus* in the ting-variant is unexpected because it is a xerophylic fungus, reported to be associated with a traditional Vietnamese alcohol starter culture known as banh men, produced from raw rice dough, water, spices, and herbs, and in traditional sausages from the islands of Cyprus as well as Mytilini [136,137]. The ting-variant is a thicker slurry compared to traditional ting, and the prevalence of this fungus could be due to the low water activity of the product. The endophyte *Epicoccum italicum* was also detected in the ting-variant produced from maize and sorghum [138]. Although previous reports show that *E. italicum* can contaminate milk-based food products, it was absent in amasi in this study [139,140]. *Cladosporium basi-inflatum* is a fungus that is part of the *Cladosporium* genus, ubiquitous in the environment [141]. Species from the *Cladosporium cladosporioides* complex were previously reported to cause infections and human health problems [142]. However, *Cladosporium* species from the air and grapevine fruit were used to produce enzyme cocktails from orange peels [143]. The presence of *C. basi-inflatum* in the ting-variant aligns with a previous report whereby this fungus was detected in barley [144]. The *Lecanicillium* species are pathogens or parasites of arthropods, nematodes, and other fungi, which have been isolated from various environments such as feces, soil, animals, and fungal material [145,146,147]. Indeed, *Lecanicillium coprophilum* was identified as a pathogen for the medicinal and edible fungus *Cordyceps militaris* as well as in the feces of *Marmota monax* [147,148]. Some antimicrobial activity against rice pathogens was observed for *L. coprophilum,* and in another study, this fungus was used for polyethylene degradation, indicating potential for industrial or bioremediation applications [149,150]. The presence of *L. coprophilum* in this study may be due to environmental contamination or an indicator of the quality of the substrates used, but further research is required. Figure 6 further highlights that the yeast *M. restricta* was dominant in amasi, followed by *C. hainanensis*, *Byssochlamys* sp., *Paecilomyces maximus,* and *Aspergillus versicolor,* which is a filamentous fungi found in food, clinical materials, soil, caves, marine or hypersaline ecosystems, as well as indoor environments [151,152]. The presence of *A. versicolor* indicates fungal contamination because *Aspergillus* is a spoilage mold found in dairy products such as cheese, pasteurized milk, raw milk, and amasi [75,153]. The presence of *Aspergillus* sp. compromises the safety of traditional fermented food products due to mycotoxin production. The presence of this fungus in the ting-variant also aligns with the previous literature, whereby the *Aspergillus* genus was identified in sorghum malts and cereal-based fermented products [154,155,156]. The yeast-like fungus, *Magnusiomyces clavatus,* was only detected in amasi in this study. This fungus is found in nature, nutrient-rich or sugar-rich sources such as industrial effluent, rotting fruit, and dairy [157,158]. Furthermore, this genus has been isolated from clinical environments and the gastrointestinal tract and is a human pathogen [159]. The presence of *M. clavatus* in amasi highlights the risks associated with spontaneous fermentations and the need for starter culture, as well as quality milk to reduce food harm. The lowest fungal diversity was observed in mahewu compared to the ting-variant and the amasi (Figure 6). This may be because milk and malted sorghum are nutrient-rich substrates that facilitate the growth of a wider range of microorganisms, whereas maize fermentations are mainly driven by lactic acid bacteria, which reduce pH, produce antioxidants, and may produce antimicrobial compounds that inhibit fungi [40,160].

### 3.9. Differentiation PCoA Analysis of Fungal Communities in Fermented Products

The principal coordinate analysis (PCoA) of Bray–Curtis dissimilarity and Venn diagram analysis in Figure 7 provide complementary insights into the structure and sharing of fungal communities across the three spontaneously fermented products. The PCoA ordination in Figure 7a reveals pronounced beta-diversity, with complete separation of the three samples along the first two axes (explaining 72.8% and 27.2% of variation, respectively). The ting-variant is positioned distinctly from amasi and mahewu, while the latter two show partial proximity but remain resolved. This strong partitioning indicates that fungal community assembly is highly substrate-dependent and indicates that the additional cooking step in the ting-variant impacted the fungal community compared to the other two spontaneously fermented products produced via traditional techniques. The Venn diagram in Figure 7b further elucidates taxonomic overlap, identifying a core mycobiota of seven taxa shared across all three products. This moderate shared component likely represents environmentally ubiquitous or resilient fungi capable of persisting across diverse fermentation matrices. Pairwise overlaps are limited (T ∩ A: 3 taxa; T ∩ M: 1 taxon; A ∩ M: 1 taxon), while unique taxa are distributed as follows: five in the ting-variant, three in amasi, and one in mahewu. The higher number of unique taxa in the ting-variant suggests greater ecological opportunity for specialized fungi, potentially introduced via grain storage or the two-stage processing (boiling and re-fermentation). In contrast, mahewu’s single unique taxon reflects a more restrictive niche, consistent with rapid acidification and anerobic conditions that limit fungal establishment. Amasi’s intermediate uniqueness aligns with spontaneous milk fermentations [44].

### 3.10. Integration of FTIR Biochemical Profiles with Microbial Community Composition

The ATR-FTIR spectral signatures presented in Figure 1 are directly consistent with the microbial community structures revealed by amplicon sequencing. In amasi, the prominent amide I/II bands (~1650 and 1540 cm^−1^) and lipid C–H stretching vibrations (~2920 and 2850 cm^−1^) align with the dominance of *Lactococcus* (50.9%) and *Enterococcus* (20.8%), which are taxa well-documented for proteolytic and lipolytic activities during milk fermentation that release peptides and free fatty acids. The shoulder at ~1740 cm^−1^, indicative of carboxylic acids and esters, corresponds to lactic acid production by LAB. In contrast, the cereal-based products (mahewu and ting-variant) exhibited intense C–O–C and C–OH polysaccharide bands (~1100–1030 cm^−1^), which reflect residual starch and exopolysaccharides produced by the highly dominant *Weissella* (98.4% in ting-variant). The strong C=O stretching vibrations (~1650–1640 cm^−1^) in both cereal ferments are consistent with the production of short-chain organic acids by *Clostridium sensu stricto* (78.3% in mahewu) and *Weissella,* genera known to drive heterofermentative metabolism and acidification. These integrated patterns demonstrate that substrate-driven microbial selection directly shapes the biochemical fingerprint of each fermented product.

### 3.11. Study Limitations and Future Directions

While this study provides a comprehensive amplicon-based snapshot of bacterial and fungal communities in three spontaneously fermented Southern African products, several limitations warrant consideration. Although FT-IR is a high-throughput fingerprinting technique for analyzing fermented products, spectral analyses should be coupled with pH and titratable acidity measurements with enough replicates to generate robust data. Furthermore, validation through targeted metabolomics, such as gas chromatography–mass spectrometry (GC-MS) for short-chain fatty acids (SCFAs) or High-Performance Liquid Chromatography (HPLC) for organic acids, would also confirm inferences made from spectral data. Furthermore, metabolomics data can be incorporated into machine learning technologies for predictive fingerprinting to determine the identity, safety, and functionality of fermented foods. Additionally, although several taxa with known pathogenic or toxigenic potential were detected at notable relative abundances, the amplicon sequencing approach used here cannot distinguish between viable and non-viable cells, nor does it provide absolute quantification or confirm the actual production of toxins or mycotoxins. Future studies should therefore incorporate culture-dependent enumeration (CFU), viability PCR (such as PMA-qPCR or v-PCR), absolute quantification methods such as digital PCR, and targeted chemical analyses using ELISA or LC-MS for mycotoxins, enterotoxins, or other microbial metabolites, to fully assess the safety risks associated with these spontaneously fermented products. Importantly, functional and safety conclusions drawn from taxonomic profiles alone remain inferential. The amplicon-based approach employed here does not provide direct evidence of functional gene content or metabolite production. Future studies should therefore incorporate shotgun metagenomics to obtain strain-level resolution and direct assessment of metabolic pathways, virulence factors, and antimicrobial resistance genes. Additionally, fungal ITS read counts were markedly lower in mahewu compared to the ting-variant and amasi, potentially introducing sampling bias and underrepresenting rare taxa in this highly acidic, low-diversity environment. The compositing of technical triplicates into single libraries per product, while reducing extraction bias, precluded formal statistical testing of differential abundance or beta-diversity (such as PERMANOVA or ANCOM-BC), limiting inference to descriptive patterns. Future studies could address these constraints by incorporating independent replicates for robust statistical analyses, shotgun metagenomics for strain-level resolution and direct functional gene detection, and multi-omics integration (such as metabolomics, volatilomics) to validate predicted pathways and elucidate metabolite–microbe interactions. Such approaches would further illuminate the ecological drivers of spontaneous fermentation in amasi, ting-type gruels, and mahewu, supporting efforts to enhance safety, consistency, and nutritional value in these culturally vital foods.

## 4. Conclusions

The present exploratory metagenomic investigation of three spontaneously fermented Southern African traditional products, amasi, ting-type cereal gruel, and mahewu, provides preliminary evidence of pronounced substrate-driven partitioning of bacterial and fungal communities, accompanied by differences in acidification and biochemical signatures detected by FTIR spectroscopy. These patterns suggest that raw material composition and spontaneous fermentation may shape microbial succession, metabolite profiles, and product characteristics, aligning with broader patterns observed in African fermented foods where cereal substrates tend to favor anerobic and plant-associated taxa, while dairy matrices appear to promote proteolysis and environmental opportunists. The recurrent detection of potentially pathogenic or toxigenic taxa highlights possible safety vulnerabilities in uncontrolled fermentations and generates hypotheses regarding the need for improved hygiene practices and the development of defined starter cultures. Conversely, the prevalence of lactic acid bacteria (LAB) previoused reported as exhibiting traits suggestive of probiotic potential and antimicrobial metabolism points towards promising opportunities for isolating beneficial strains that could enhance the nutritional value and shelf life of traditional southern African fermented products. Overall, these findings generate several testable hypotheses concerning the ecological drivers of spontaneous fermentation in these culturally important foods. Future research integrating shotgun metagenomics, metabolomics, and sensory evaluation will be essential to validate functional predictions, explore strain-level variations, and support the preservation and modernisation of these culturally significant foods. Future studies should incorporate shotgun metagenomics to provide strain-level resolution and direct insights into functional genes and metabolic pathways. In addition, targeted and untargeted metabolomics analyses should be performed in parallel and integrated with the microbial community data through correlation analyses and multi-omics modelling to establish direct linkages between specific taxa and key metabolites. Furthermore, microbial source tracking approaches such as SourceTracker or FEAST are recommended to differentiate the contributions of microorganisms originating from the raw substrates versus the processing environment and equipment.

## Figures and Tables

**Figure 1:**
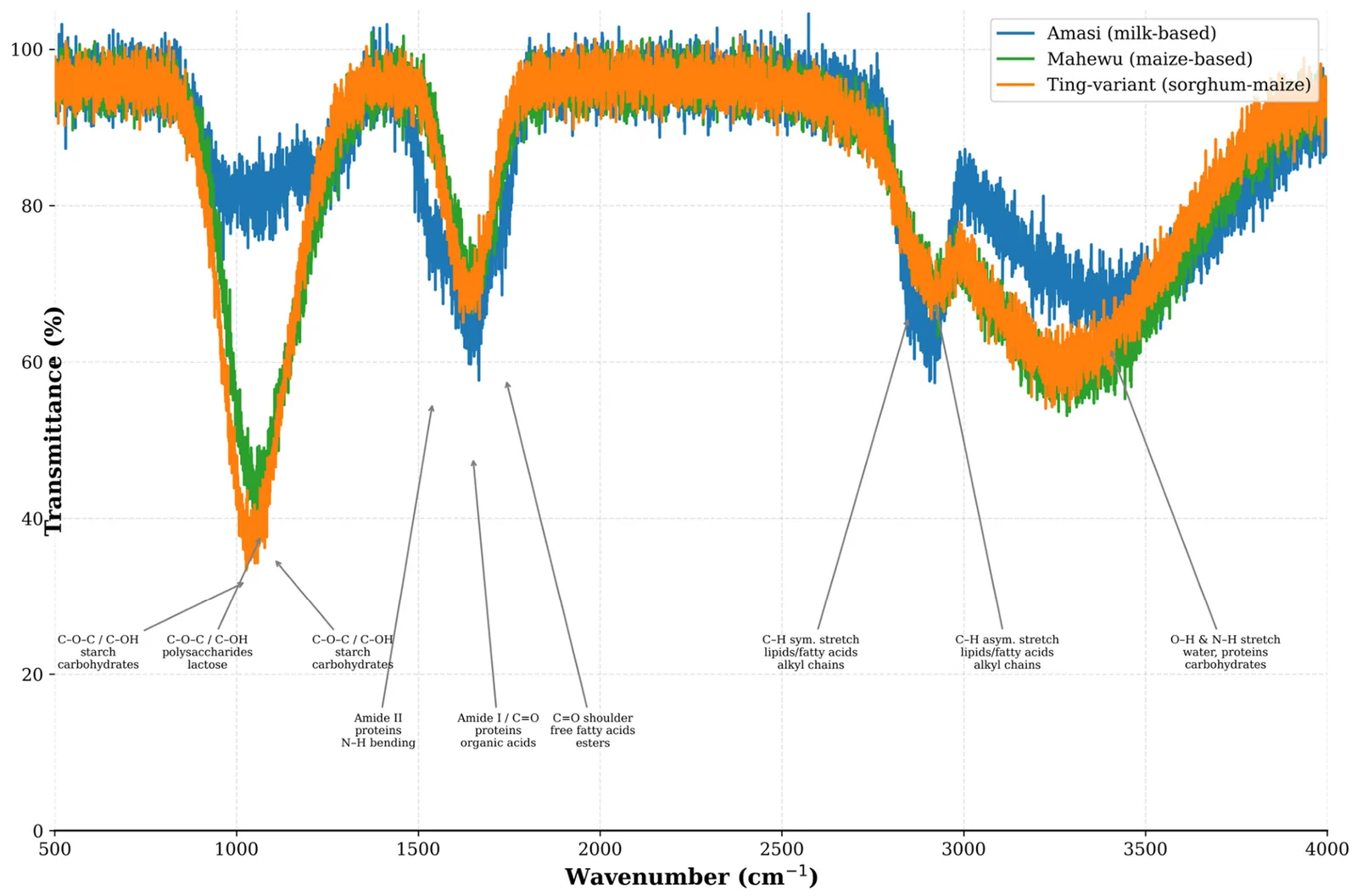
FTIR spectra of three spontaneously fermented Southern African traditional products. Transmittance spectra (4000–500 cm^−1^) of fermented products obtained using ATR-FTIR spectroscopy with 16 co-a scans per sample. Blue band (amasi); green band (mahewu); orange band (ting-variant).

**Figure 2:**
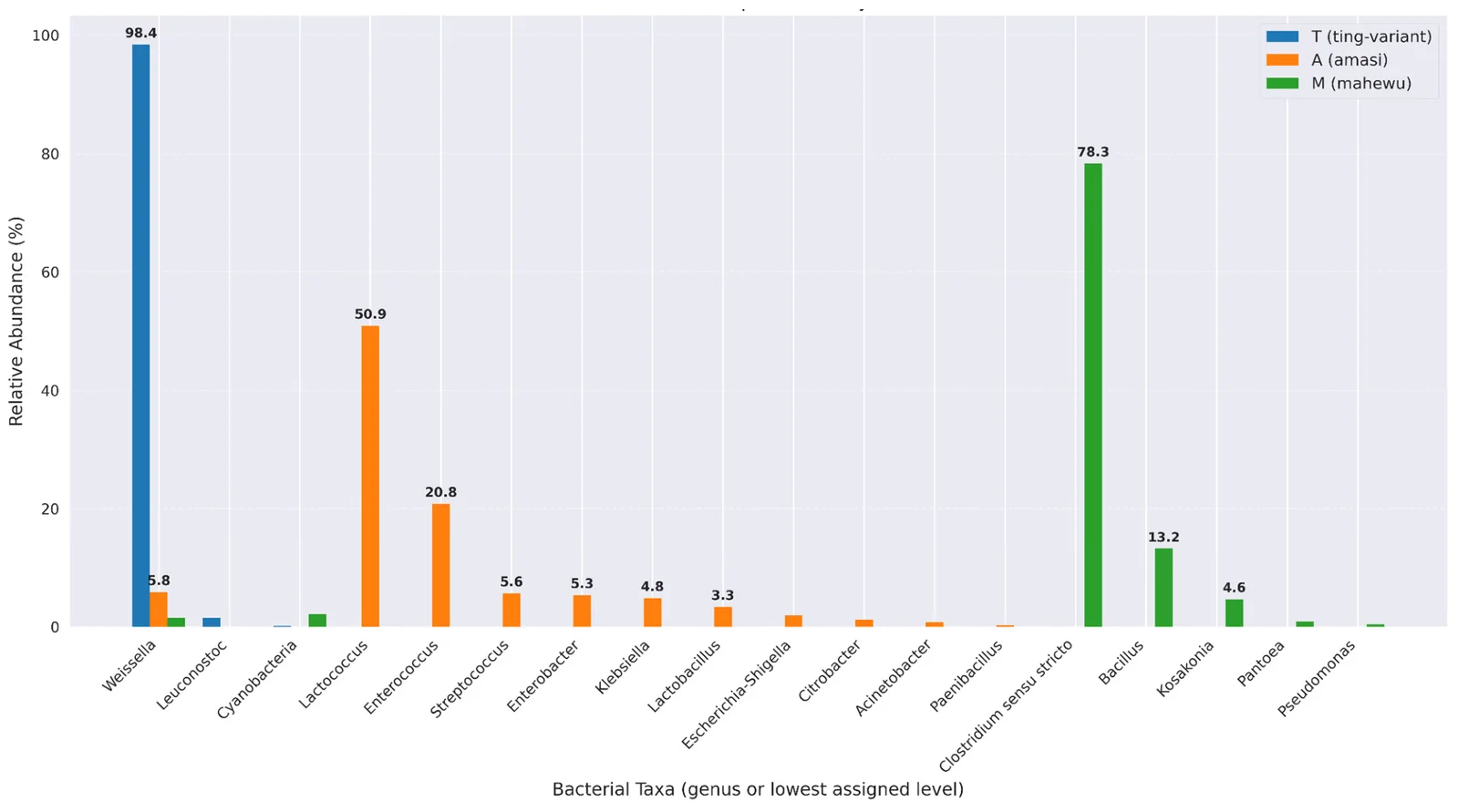
Relative abundance of bacterial taxa in spontaneously fermented Southern African traditional products. Stacked bar chart showing the relative abundance (%) of bacterial genera (or lowest confidently assigned taxonomic level) in the three products: T (ting-variant, sorghum- and maize-meal-based cereal gruel), A (amasi, fermented cow’s milk), and M (mahewu, maize-based cereal gruel). Taxa are ordered from highest to lowest abundance for visual comparison.

**Figure 3:**
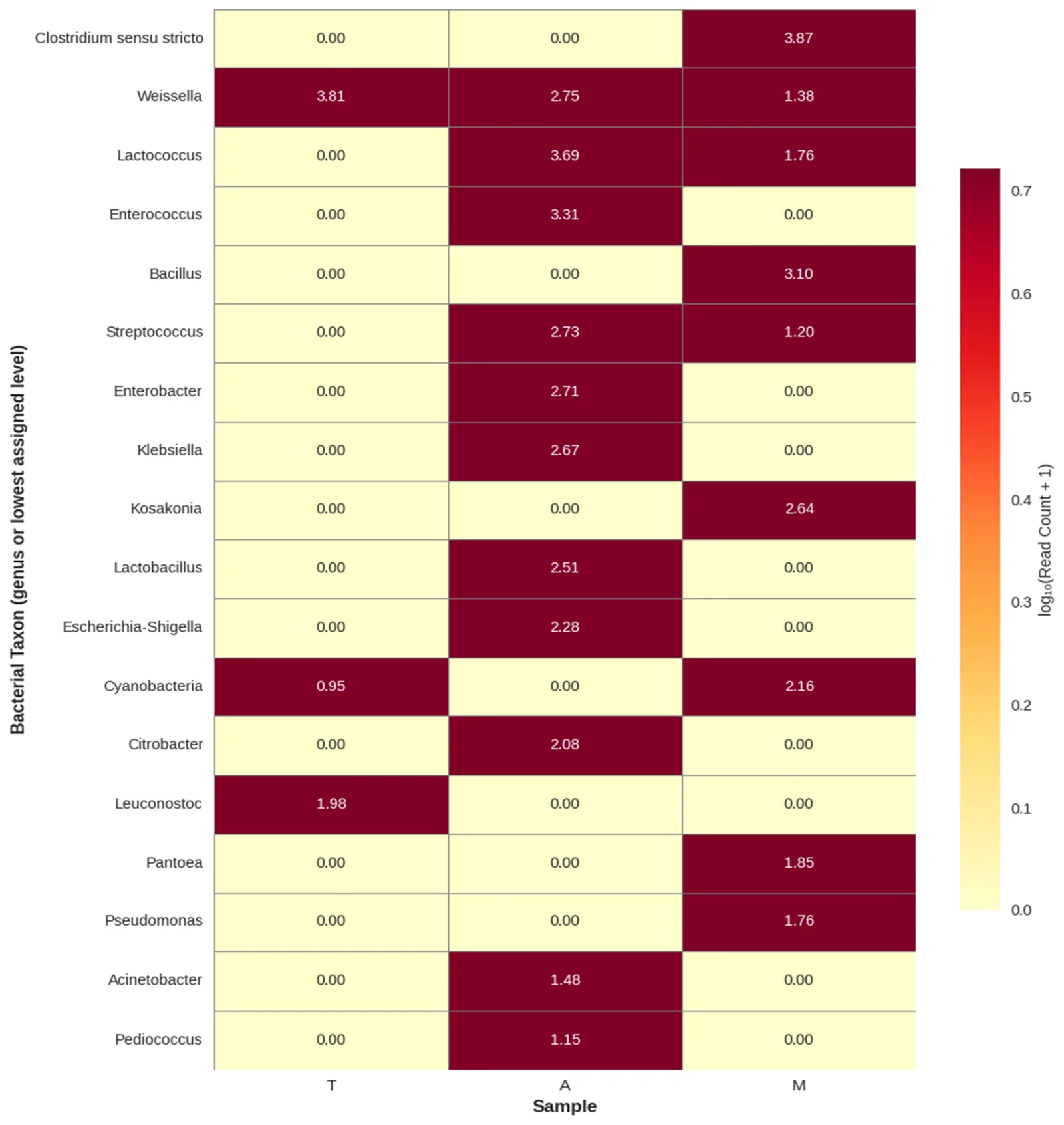
Taxonomic heatmap of bacterial communities in spontaneously fermented Southern African products. Log_10_-transformed read counts (log_10_[read count + 1]) of bacterial taxa (genus or lowest confidently assigned taxonomic level) across the three products: T (ting-variant), A (amasi), and M (mahewu). Color intensity indicates abundance, with warmer (darker red) tones representing higher log-transformed counts.

**Figure 4:**
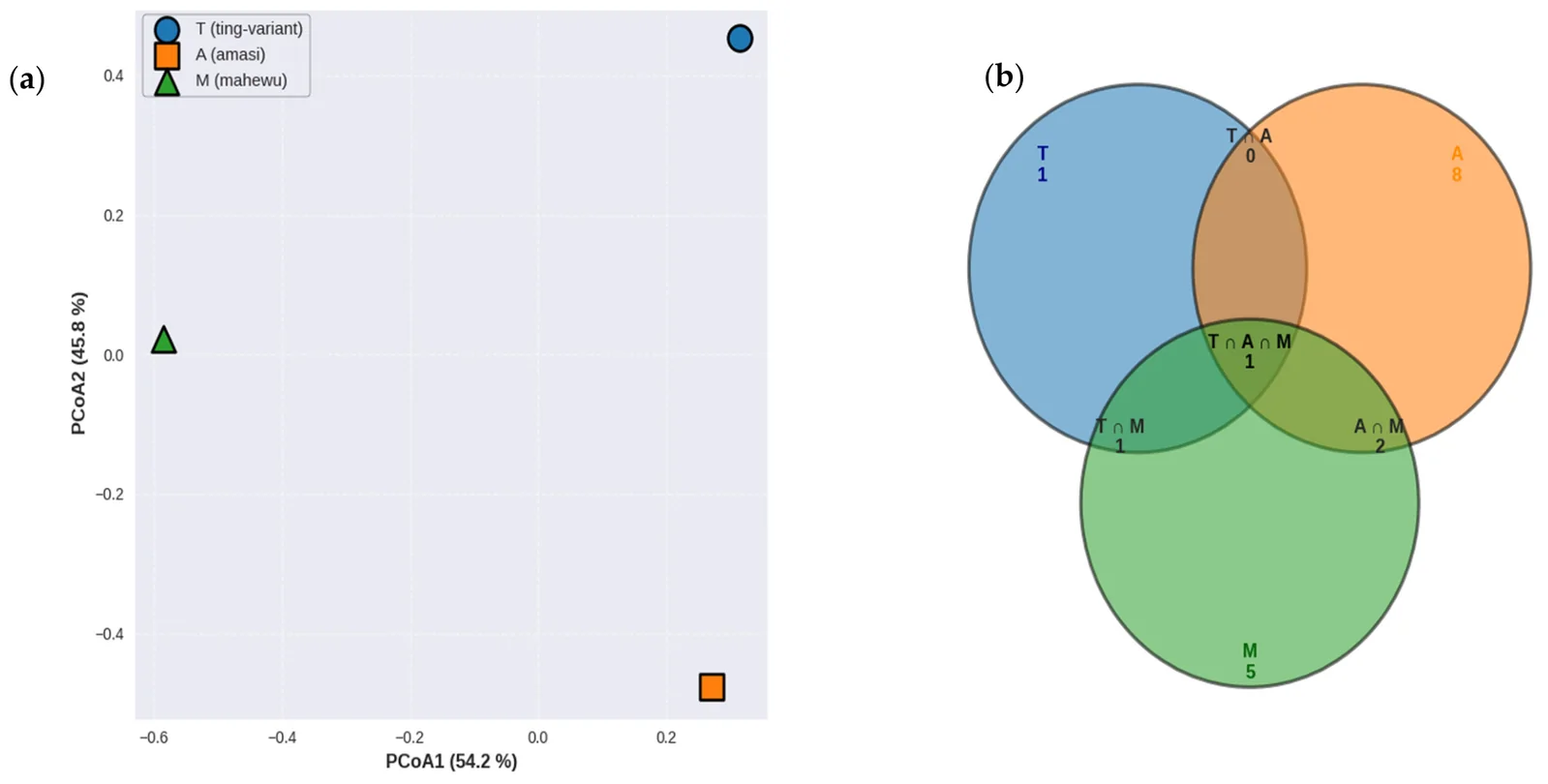
(**a**) Principal coordinate analysis (PCoA) based on Bray–Curtis dissimilarity of bacterial communities in the three products: T (ting-variant), A (amasi), and M (mahewu). PCoA1 and PCoA2 explain 54.2% and 45.8% of the total variation, respectively. (**b**) Venn diagram demonstrating beta-diversity and shared bacterial taxa in spontaneously fermented Southern African products.

**Figure 5:**
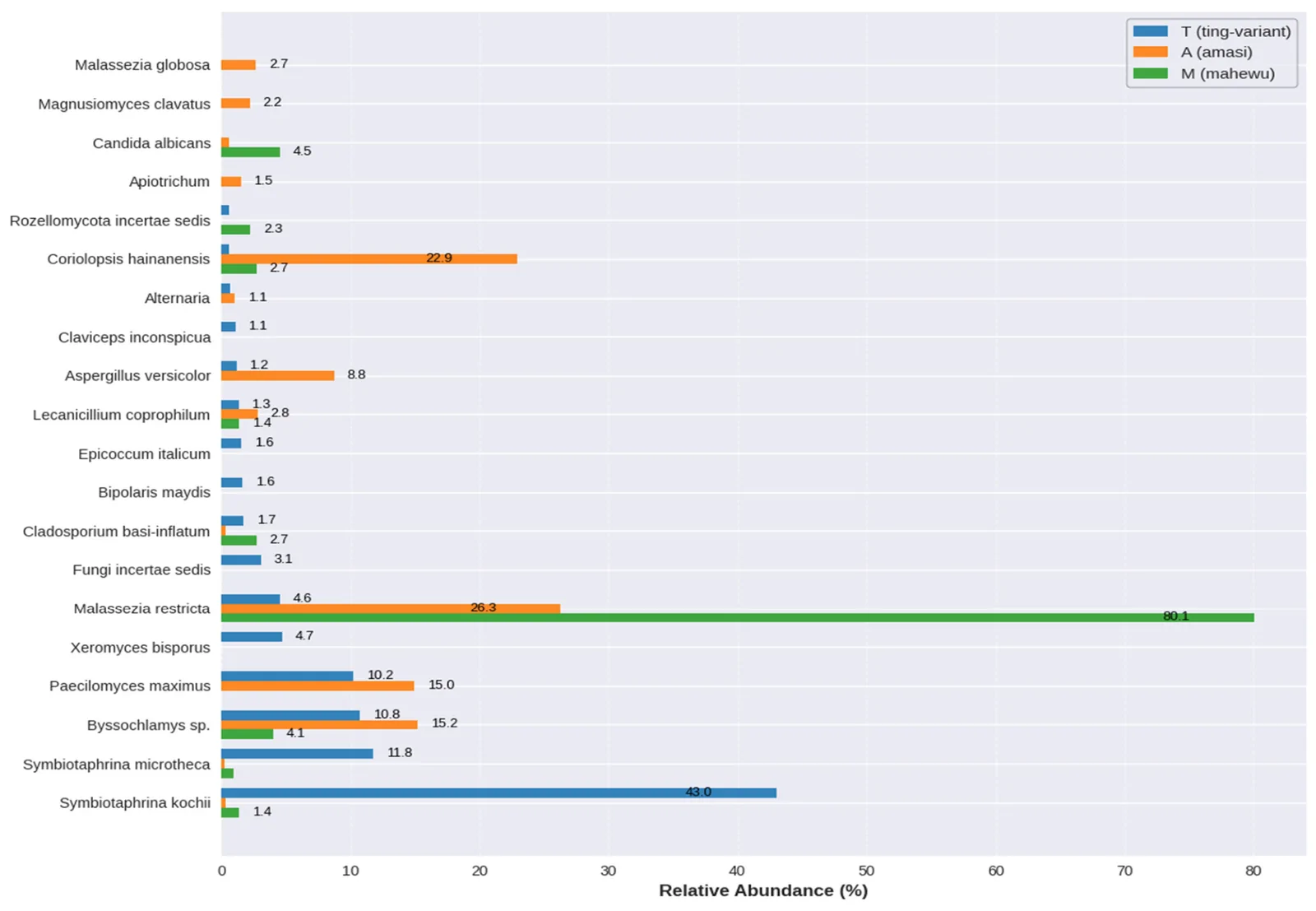
Relative abundance of fungal taxa in spontaneously fermented Southern African products. Horizontal bar chart showing the relative abundance (%) of fungal taxa (genus/species or lowest assigned level) from PacBio ITS sequencing data. Products: T (ting-variant, sorghum- and maize-meal-based cereal gruel), A (amasi, fermented cow’s milk), and M (mahewu, maize-based cereal gruel). Taxa are ordered from highest to lowest abundance for visual comparison.

**Figure 6:**
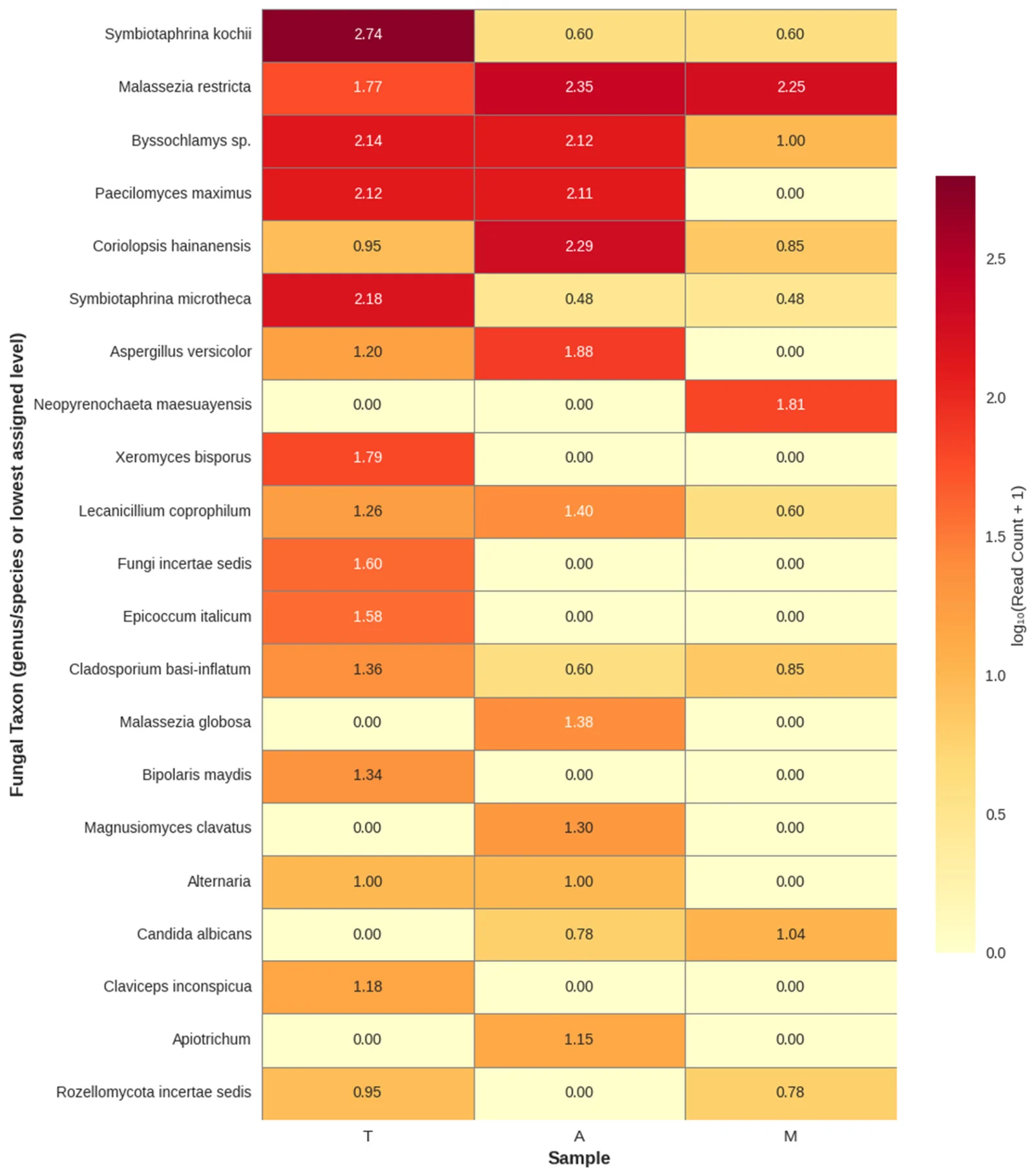
Taxonomic heatmap of fungal communities in spontaneously fermented Southern African products. Log_10_-transformed read counts (log_10_[read count + 1]) from PacBio ITS sequencing, showing fungal taxa (genus/species or lowest assigned level) across T (ting-variant), A (amasi), and M (mahewu). Warmer colors indicate higher enrichment.

**Figure 7:**
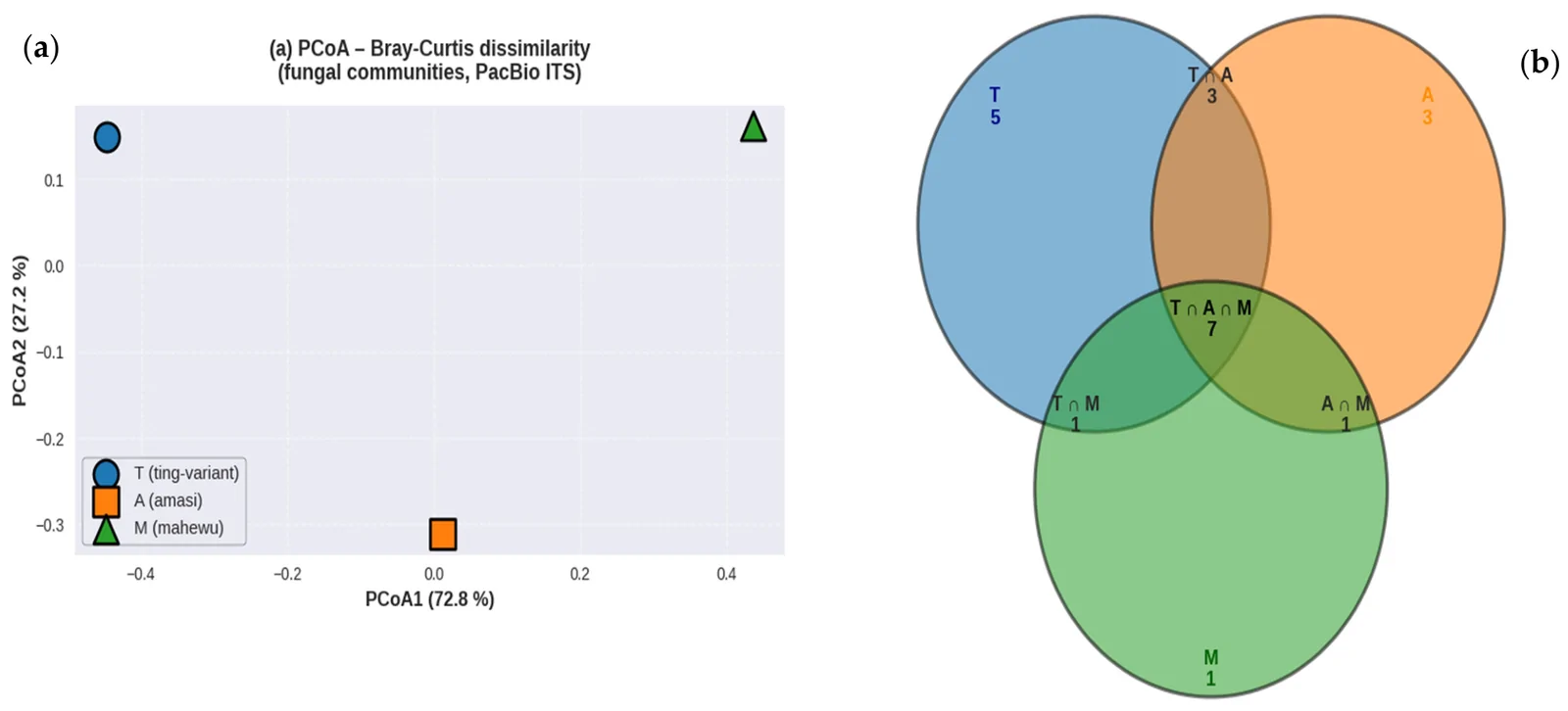
Beta-diversity and taxonomic overlap of fungal communities in spontaneously fermented Southern African products. (**a**) Principal coordinate analysis (PCoA) based on Bray–Curtis dissimilarity of fungal ITS amplicon data (PacBio sequencing). Axes explain 72.8% (PCoA1) and 27.2% (PCoA2) of variation, showing complete separation of T (ting-variant), A (amasi), and M (mahewu). (**b**) Venn diagram depicting shared and unique fungal taxa (presence/absence at genus/species or lowest assigned level).

**Table 1:** pH values of the three spontaneously fermented Southern African traditional products after 48 h of fermentation.

Fermented Product	pH
Amasi	4.348
Mahewu	4.005
Ting-variant	3.451

## Data Availability

The original contributions presented in this study are included in the article/Supplementary Materials. Further inquiries can be directed to the corresponding author.

## Supplementary Materials

The following supporting information can be downloaded at: https://www.mdpi.com/article/10.3390/foods15213935/s1, Figure S1: Alpha diversity of the bacterial and fungal communities in Southern African traditional fermented foods; Figure S2: (a) Functional heatmap–predicted KEGG pathway abundances (%); (b) The relative contribution of key functional categories of predicted KEGG pathway abundances. Figure S3: Rarefaction curves of the bacterial and fungal communities of traditional fermented foods.
