# Supplementary material for: Exploratory Amplicon-Based Metagenomic Profiling and Chemical Fingerprinting of Three Spontaneously Fermented Southern African Traditional Products
Source: Foods. 2026 Nov 3;15(21):3935. doi: 10.3390/foods15213935 (PMC13762676; doi:10.3390/foods15213935)
Supplement: Supplementary file 1 [file foods-15-03935-s001.zip › foods-4256546-supplementary.pdf]

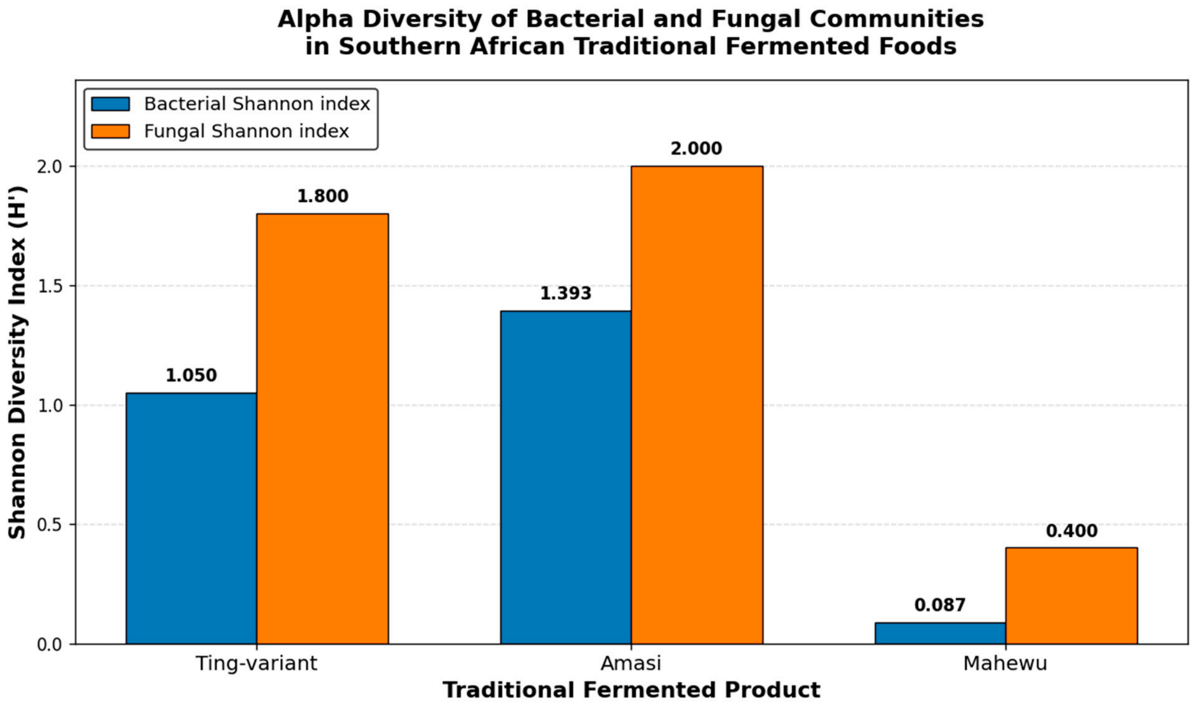

Figure S1: Alpha diversity of the bacterial and fungal communities in Southern African traditional fermented foods.

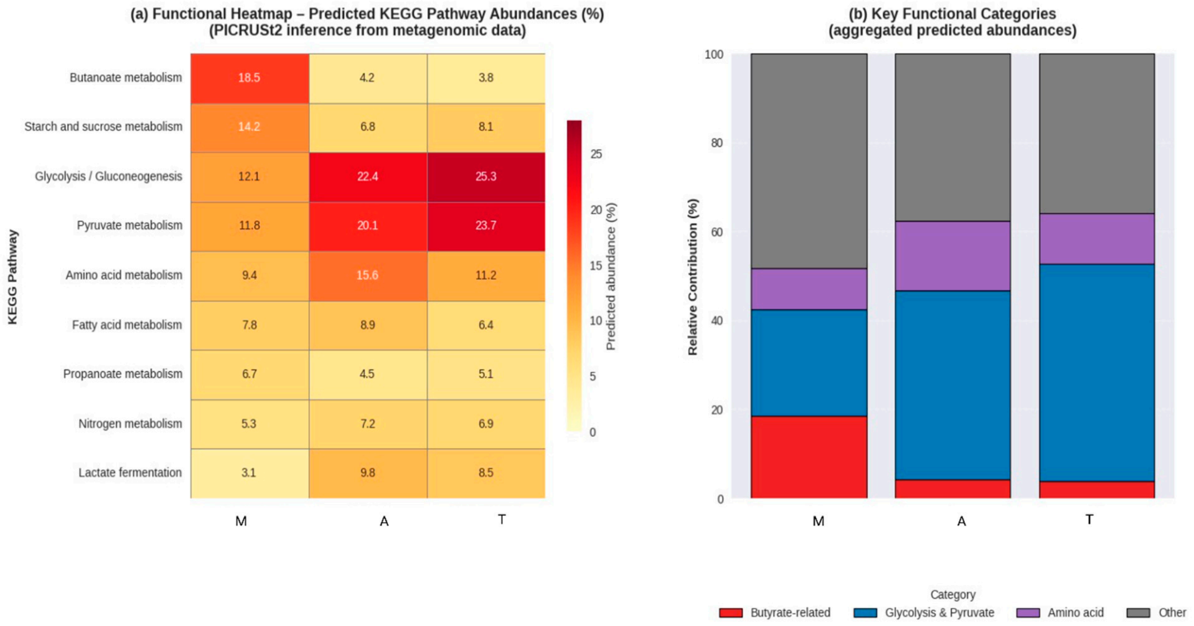

Figure S2: (a) Functional heatmap–predicted KEGG pathway abundances (%); (b) The relative contribution of key functional categories of predicted KEGG pathway abundances.

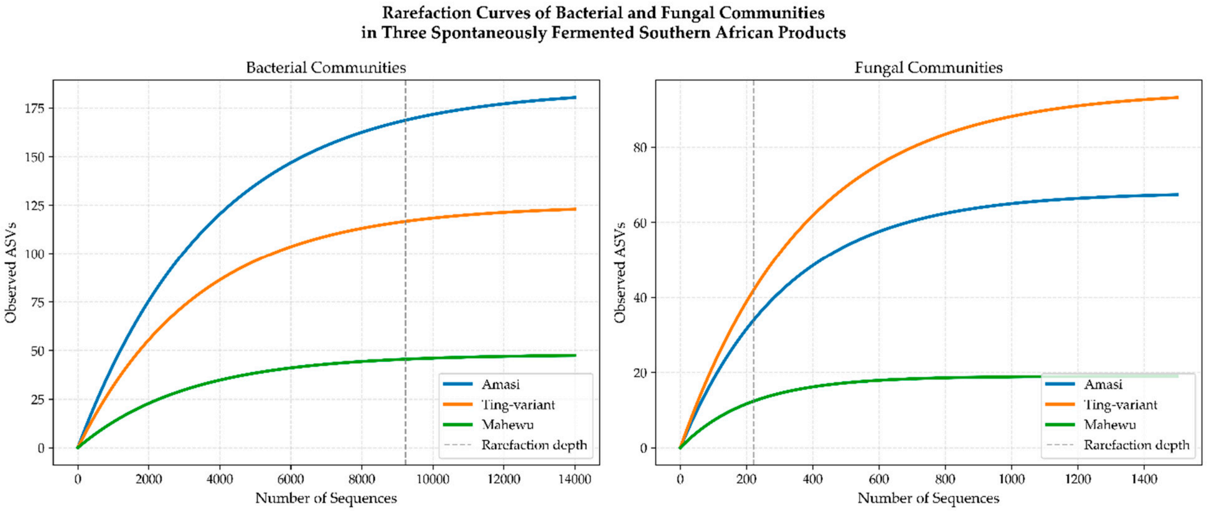

Figure S3: Rarefaction curves of the bacterial and fungal communities of traditional fermented foods.
